# HIV-1 Tat-induced disruption of epithelial junctions and epithelial-mesenchymal transition of oral and genital epithelial cells lead to increased invasiveness of neoplastic cells and the spread of herpes simplex virus and cytomegalovirus

**DOI:** 10.3389/fimmu.2025.1541532

**Published:** 2025-02-13

**Authors:** Sharof Tugizov

**Affiliations:** Department of Medicine, School of Medicine, University of California, San Francisco, San Francisco, CA, United States

**Keywords:** human immunodeficiency virus, Tat, mucosal epithelium, disruption of epithelial junctions, epithelial-mesenchymal transition

## Abstract

Human immunodeficiency virus (HIV-1) transactivator Tat is a unique multi-functional viral protein secreted by infected cells. Although its primary function is to promote HIV-1 transcription, secreted Tat interacts with neighboring cells and induces numerous disease-associated pathological changes. Despite the substantial reduction of viral load and disease burden, Tat expression and secretion persist in people living with HIV who are undergoing treatment with highly effective combination antiretroviral therapy (cART). Tat interacts with both oral and genital epithelial cells and impairs their mucosal barrier functions, which facilitates the entry of other pathogenic viruses. Tat-mediated interactions with both human papillomavirus (HPV) -infected and HPV-negative neoplastic epithelial cells lead to epithelial-mesenchymal transition and increased invasiveness of malignant cells. Likewise, Tat-induced disruption of oral epithelial cell junctions leads to herpes simplex virus-1 (HSV-1) infection and spread via exposure of its receptor, nectin-1. HIV-1 Tat facilitates infection and spread of human cytomegalovirus (HCMV) by activating mitogen-activated protein kinases (MAPK) and promoting NF-κB signaling, both critical for the replication and production of progeny virions. HIV extracellular Tat also plays a critical role in human herpesvirus 8 (HHV8) -caused Kaposi sarcoma (KS) pathogenesis by synergizing with HHV-8 lytic proteins and promoting the proliferation, angiogenesis, and migration of endothelial cells. Collectively, these findings emphasize the critical impact of HIV-1 Tat on HIV/AIDS pathogenesis during the cART era and highlight the need for further research on the molecular mechanisms underlying Tat-mediated interactions with oral and genital mucosal epithelial cells.

## 1 Introduction

The HIV-1 trans-activator of transcription protein, Tat, regulates the viral promoter and is responsible for transcriptional elongation, which is critical for HIV replication. Tat is also involved in regulating the expression of cellular genes and key signaling pathways, which are critical for the production and spread of HIV. Moreover, HIV-1 Tat is continuously secreted into the blood circulation of HIV-1-suppressed individuals and may reach various tissues and organs leading to their dysfunction.

HIV coinfection is common with several pathogenic human viruses, including high-risk HPV (1–4), HSV-1 and -2 (5–9), HCMV (10–12), Kaposi’s sarcoma-associated herpesvirus (KSHV) (13–15) and hepatitis C virus (HCV) (16–19).

Accumulating evidence supports that interactions of Tat with oral and genital mucosal epithelial cells disrupt their intercellular junctions and facilitate the spread of pathogenic human viruses, including HPV, HSV, and HCMV. Moreover, Tat may induce epithelial-mesenchymal transition (EMT), which is critical for the invasion of HPV-infected neoplastic epithelial cells. Tat plays a critical role in the development of Kaposi’s sarcoma (KS) by promoting proliferation, angiogenesis, and/or migration of target endothelial cells. The focus of this mini-review is to discuss the interaction of Tat with oral and genital mucosal epithelial and endothelial cells and to elucidate its role in the impairment of the mucosa barrier and induction of EMT, factors that are critical for the spread of viruses and acceleration of neoplastic processes, respectively.

## 2 HIV-1 Tat: structure and function

HIV-1 Tat is a small (14-16 kDa) protein encoded by the viral genome that includes several important functional domains (20–23). The two forms of Tat generated by alternative splicing include one generated from a two-exon transcript that varies in length from 86 to 101 amino acids (aa) depending on the specific viral isolate. The second form of Tat is translated from a singly spliced one-exon transcript and is 72 aa long. The Tat amino-terminal domain (aa 1-48) contains a cysteine-rich motif that is responsible for activating HIV-1 transcription via its binding to a transactivation of response (TAR) element of the newly transcribed viral genomic RNA (24). The arginine-rich basic domain of Tat (aa 49-58) plays a critical role in its nuclear localization and binding to the TAR element (25). The basic domain of Tat also contains a protein transduction domain (PTD) which is a specific motif, YGRKKRRQRRR that mediates Tat internalization by bystander cells via interactions with heparan sulfate proteoglycans (HSPGs) (26–33). The Tat glutamine (Q)-rich domain (aa 60-72) also contributes to its interaction with the TAR element and mediates Tat-induced apoptosis (34, 35). HIV-1 Tat also contains a carboxy-terminal (aa 65-80) arginine, glycine, and aspartic acid (RGD) motif that facilitates its binding to various integrins, including αvβ1, αvβ3, and α5β1 (36–40).

## 3 HIV-1 Tat: expression and secretion in HIV-1 suppressed individuals

One of the important features of HIV-1 Tat is its efficient secretion from virus-infected cells (30, 41–46) Tat crosses the plasma membrane via unconventional pathways that do not involve the endoplasmic reticulum and/or the Golgi/trans-Golgi network (43). Although the molecular mechanisms underlying Tat secretion remain unclear, secretion involves Tat binding to phosphatidylinositol-4,5-bisphosphate (PtdIns(4,5)P_2_) located in the inner leaflet of the plasma membrane, an interaction mediated by the basic domain of Tat (aa 48–57) and a conserved tryptophan (aa 11) (47). This interaction initiates oligomerization-mediated pore formation in the membrane, followed by Tat translocation and incorporation into exosomes, which mediate Tat’s secretion (22, 43, 44, 47–50). Secretion may also require Tat interactions with three short, non-consecutive cytoplasmic loops at the carboxy-terminus of the cellular Na^+^, K^+^-ATPase pump alpha subunit (51).

HIV-1-infected lymphocytes, macrophages, and dendritic cells/Langerhans cells (DC/LCs), as well as cell-free virus and viral RNA/DNA, have all been detected in oropharyngeal and genital mucosal epithelia and in salivary, and cervicovaginal secretions of people living with HIV (PLWH) who were undergoing combination antiretroviral therapy (cART) as well as those who were not (42, 52–66). HIV-1 Tat-positive CD4^+^ lymphocytes, macrophages, and DC/LCs were detected in the oropharyngeal and anogenital mucosal epithelia of PLWH, including those undergoing treatment with cART (42).

Westendorp and colleagues (67) were the first to demonstrate HIV-1 Tat secretion into the bloodstream; this finding has since been confirmed by others (68, 69). In pre-cART era, serum Tat concentrations in PLWH typically ranged from 0.1-40 ng/mL, sometimes reaching levels as high as 250-550 ng/mL (70–74). HIV-1 Tat was also detected in the serum of PLWH who achieved viral suppression with cART, where Tat concentration ranged from 0 to 14 ng/ml (46). Tat was detected in 25% of cART-treated individuals (46). The serum concentrations of Tat during early cART and late cART were 2-40 ng/ml and 0.2-9 ng/ml, respectively (68, 70).

HIV-1 Tat was also detected in the blood-cerebrospinal fluid of 37% of PLWH maintained on a long cART regimen (75, 76) at concentrations in the 0.2-6.5 ng/mL range. Functional Tat was detected in exosomes from 34.4% of these individuals (75). Secretion of Tat was detected in the saliva of ART-treated and -untreated individuals (42).

Tat was detected in brain and cerebrospinal fluid in the absence of virus replication (73). Furthermore, in experiments performed *in vitro*, Tat was expressed and secreted from HIV-infected cells treated with protease inhibitors that inhibited viral replication, indicating that Tat production and secretion requires no cleavage events catalyzed by the viral protease (73, 77). Thus, Tat may continue to be expressed and released in the presence of a protease inhibitor, which is among the critical components of cART (73, 75, 77). Tat/TAR-containing exosomes can be endocytosed by HIV-1-infected cells that contain an inactive (latent) provirus (75). Internalized Tat/TAR may then reactivate the latent provirus via induction of host signaling pathways such as those involving NF-κB and transactivation of the HIV-1 long terminal repeat (LTR), which may explain Tat expression detected in PLWH maintained on cART (75). Tat expression in PLWH treated with cART may also result from ongoing abortive and/or spontaneous viral transcription that promotes expression of viral early genes, including Tat (78, 79). Collectively, these findings suggest that HIV-1 Tat is produced continuously by infected cells and released into circulation despite ongoing cART and that circulating Tat may be delivered directly to tissues and organs and internalized into epithelial and other host cells.

Furthermore, cART drugs have only a limited capacity to penetrate solid tissues, including lymph nodes and epithelial tissues (80–83), Thus, HIV-1 replication may persist in these drug-inaccessible environments, leading to the expression and release of Tat and its spread within the surrounding tissues and organs. Cervicovaginal secretion of HIV-1 in ART-treated women with low to undetectable plasma viral loads suggested that there may be a cohort of local intramucosal HIV-1-infected cells that remained capable of producing virus due to lack of drug accessibility (64, 84–88). This hypothesis might also explain the persistent detection of HIV-1 in the semen of ART-treated infected men with undetectable viral loads in circulation (89). Collectively, these findings suggest that ART-inaccessible tissues may serve as a reservoir for HIV-1 and that these cells may release Tat continuously into the surrounding environment (45, 80–83).

## 4 HIV-1 Tat disrupts tight junctions of epithelial cells

**Figure 1:**
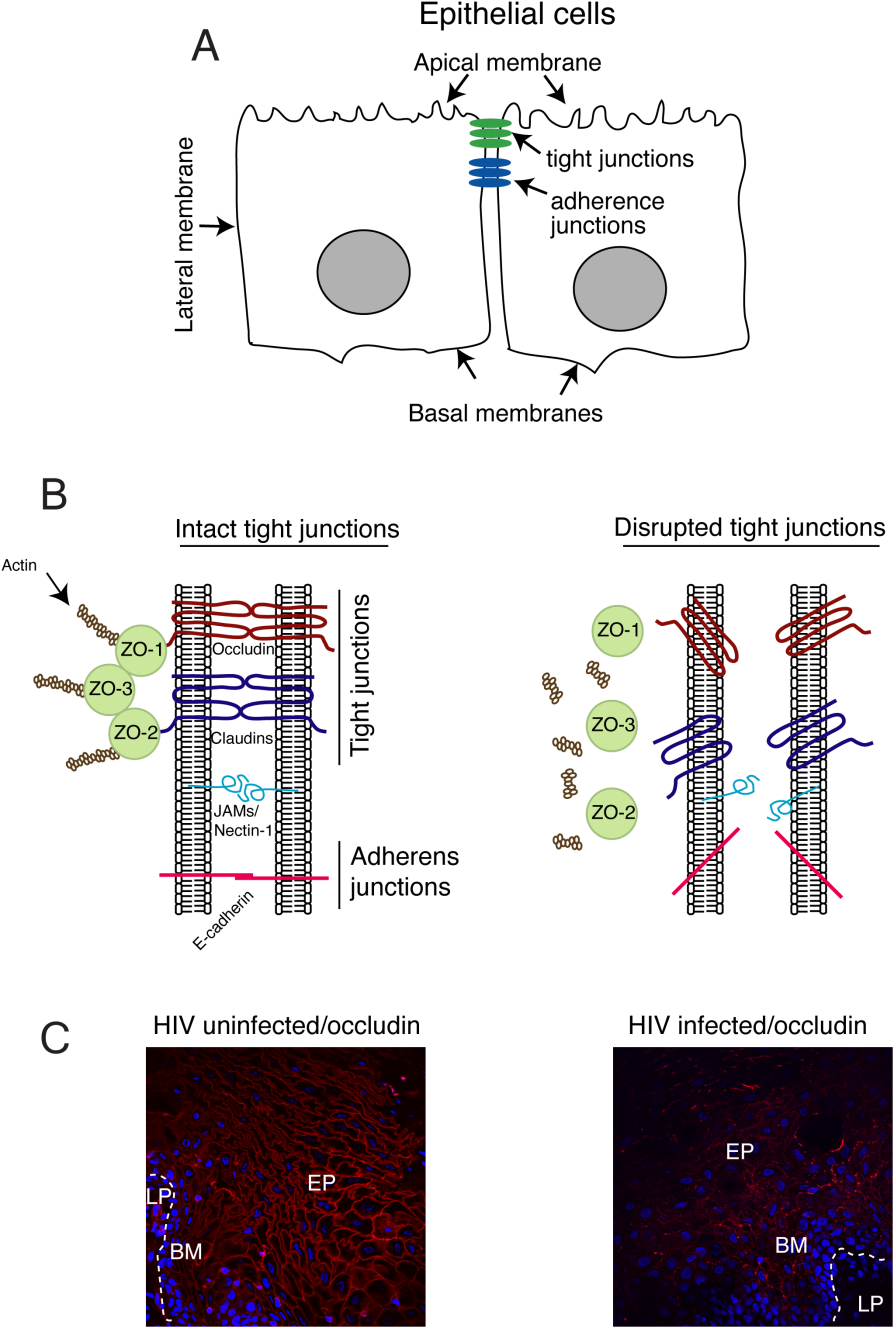
Model of HIV-1 Tat-induced disruption of tight junctions of mucosal epithelium. **(A)** Oropharyngeal and genital mucosal epithelial cells have a polarized organization with well-developed tight and adherens junctions and distinct apical and basolateral membranes. (**B**, left panel) Tight junctions are formed between epithelial cells via the lateral interaction of the transmembrane proteins, occludin and claudins, which are associated with cytoplasmic proteins ZO-1, ZO-2, and ZO-3, which provide links to the cortical actin cytoskeleton. Junctional adhesion molecule 1 (JAM-1) is also localized at the tight junctions of epithelial cells. The cell adhesion protein nectin-1 is sequestered within the adhesion junctions. (**B**, right panel) Disruption of the tight and adherens junctions of mucosal epithelium. (**C**, left panel) Buccal biopsy samples from HIV-1-infected ART-treated and HIV-1-negative individuals were immunostained for occludin (red). EP, epithelium; BM, basement membrane; LP, lamina propria. The continuous ring-shaped pattern of occludin localization on the membrane of buccal epithelial cells from HIV-1-uninfected individuals indicates the intact tight junctions of buccal mucosal epithelium. (**C**, right panel) By contrast, the absence or discontinuous/fragmented ring-shaped pattern of occludin immunostaining in buccal epithelial cells from HIV-1-infected individuals shows disrupted tight junctions of buccal mucosal epithelium. The models generated for this and all other figures to follow were created using Adobe^®^ Illustrator.

The oropharyngeal, ectocervical, vaginal, and foreskin epithelia consist of a multilayered, stratified squamous epithelium; the endocervical and intestinal mucosa is also covered with a monostratified simple epithelium. These epithelial cells form numerous intercellular junctions (42, 90–100). Among these, tight and adherens junctions are critical for maintaining the morphological and physiological features of mucosal epithelia (**Figure 1**). Tight junctions within the mucosal epithelium form an intramembranous “fence” that links neighboring cells and maintains a physical barrier that protects against external environment (101). Tight junctions are established by the transmembrane proteins known as occludin and claudins in association with the cytoplasmic proteins zonula occludens-1 (ZO-1), ZO-2, and ZO-3 (**Figure 1B**, left panel), which linked to the actin cytoskeleton (102). Junctional adhesion molecule 1 (JAM-1) is specifically localized at the tight junctions of epithelial cells and is involved in the regulation of junctional integrity and paracellular permeability (103). Intercellular adherens junctions are formed by homotypic interactions of the transmembrane protein, E-cadherin, which is connected to intracellular proteins p120 and α- and β- catenins and the actin cytoskeleton (104).

Tight junctions in oral, intestinal, and genital mucosal epithelia are disrupted in PLWH, leading to barrier impairment (**Figure 1B**, right panel, and C) (42, 105–107). Interactions of HIV-1 Tat with oral epithelial cells lead to activation of MAPK and substantial disruption of complexes containing ZO-1, occludin, and claudin-1 (42). HIV-1 glycoprotein (gp)120 also disrupts epithelial junctions; combinations of Tat and gp120 amplify HIV-1-induced impairment of mucosal epithelial barrier functions (42, 108, 109). In oral epithelial cells, HIV-1 Tat activates MAPK and NF-κB signaling, leading to the upregulation of matrix metalloproteinase (MMP)-9 expression and activity (109), and the degradation of epithelial junctions.

## 5 HIV-1 Tat induces epithelial-mesenchymal transition and amplifies the invasiveness of human papillomavirus-infected neoplastic cells

Human papillomavirus (HPV) is an oncogenic virus (110, 111). The incidence of HPV-associated oropharyngeal, cervical, and anal cancer is approximately 6-, 22-, and 80-times higher, respectively, in PLWH compared to those who remain uninfected (112–119). Although a highly effective anti-HPV vaccine is available, its value is limited in PLWH because most of these individuals have already been infected with high-risk HPVs, including HPV-16 and HPV-18.

Although HIV-1 may increase the incidence of HPV-associated cancers by attenuating immune responses, accumulating evidence has indicated that the direct interaction of HIV proteins with HPV proteins and HPV-infected epithelial cells may also play a critical role in the progression of HPV-associated malignancy (119–121).

Tat (and/or gp120) proteins disrupt tight junctions of oral and genital mucosal epithelium, thereby reducing the barrier function of mucosal epithelium and facilitating paracellular penetration of HPV-16 (42). This facilitated HPV paracellular spread through strata spinosum and granulosum layers, leading to the infection of basal/parabasal cells, the site of initiation of the HPV life cycle (122).

**Figure 2:**
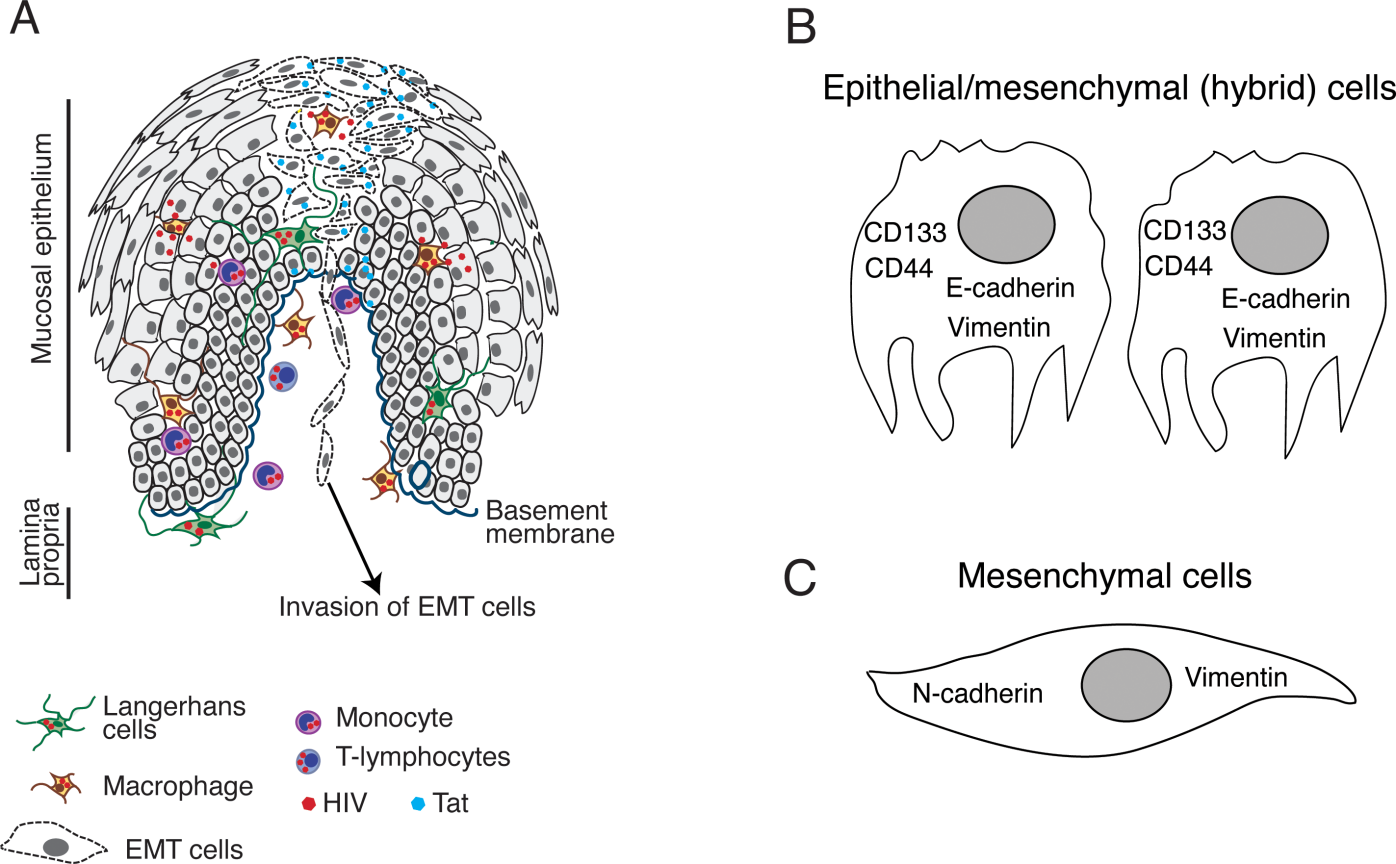
HIV-1 Tat-induced EMT in neoplastic oral and genital epithelial cells. **(A)** HIV-infected CD4^+^ lymphocytes, macrophages, and DC/LCs can migrate into the epithelium and secrete HIV-1 Tat in the HPV-infected or HPV-negative neoplastic mucosal environment (42). This will lead to the activation of MAPK and TGF-β signaling pathways followed by induction of the EMT phenotype in which epithelial cells lose intact tight and adherens junctions and **(B)** coexpress E-cadherin and vimentin. Cells displaying this hybrid EMT phenotype also express stem cell markers and become CSCs with the potential to migrate through the basement membrane and initiate invasive cancer. **(C)** Further progression of EMT may lead to the loss of E-cadherin expression and a parallel upregulation of N-cadherin and vimentin as cells acquire a mesenchymal spindle-like morphology. These cells may also be highly invasive and capable of migration.

Tat-induced activation of MAPK and transforming growth factor (TGF)-β signaling in normal and neoplastic oral and genital epithelia led to epithelial-mesenchymal transition (EMT) (120, 121), which is a physiologic process that provides critical contributions to embryonic development (123). However, EMT also promotes epithelial neoplasia, including the invasion and spread of cancer cells (124–132). EMT results in the loss of polarity of epithelial cells, which is followed by loss of adherens (E-cadherin) and tight junctions as well as critical cell-adhesive properties (**Figure 2**). While in the intermediate stages of EMT, cells may express both mesenchymal (i.e., vimentin) and epithelial (i.e., E-cadherin) markers. Cells with this hybrid phenotype may be highly invasive and contribute to the formation of cancer metastases (**Figure 2B**) (133–138). Cells displaying the hybrid phenotype will then develop a spindle cell-type shape and express additional mesenchymal markers, including vimentin, fibronectin and N-cadherin and lost E-cadherin; these cells are also highly invasive (**Figure 2C**) (133–138).

Neoplastic cells with an EMT phenotype may also express stem cell markers and become cancer stem cells (CSCs) with undifferentiated stem cell properties with the capacity for self-renewal and uncontrolled proliferation (134–143). CSCs detected in solid oropharyngeal and genital tumors express numerous stem cell markers, including CD44, CD24, CD133, aldehyde dehydrogenase 1A, CD10, CD147, CD117, CD24, podoplanin, laminin subunit beta 3, and laminin subunit gamma 2 (134–140, 142–172). Recent results suggest that the expression of one or more of these CSC markers may significantly increase resistance to drugs and apoptosis and promote survival, growth, and expansion of invasive cancers (129–131).

Activation of the TGF-β signaling pathway is critical for the induction of EMT in neoplastic cells (173, 174). The binding of the mature form of TGF-β to its receptor TGFBR2 initiates activation of Smad family transcription factors and other specific transcriptional regulators, including Slug, Twist1, and Snail. These events lead to the upregulation of N-cadherin, fibronectin, and vimentin and the downregulation of E-cadherin (175, 176) following induction of EMT.

HIV-1 Tat binding to α5β1 and αvβ3 integrins via its RGD domain increases Ras-induced ERK phosphorylation and activation of MAPK signaling (177), which is critical for upregulation of the TGF-β signaling pathway and induction of EMT (120, 121). HSPG activates TGF-β signaling by depositing latent TGF-β -binding protein-1 in the extracellular matrix, thereby enhancing the TGF-β binding to its primary receptors (178), which is also critical for EMT induction. Tat binding to HSPG may modulate the HSPG-enhanced interaction of TGF-β with its receptors (179). Although TGF-β signaling is critical for EMT induction, additional signaling pathways also contribute to this process depending on the cell type and cellular microenvironment. For example, introduction of HIV-1 Tat and gp120 to cells of the HPV-16-infected cervical cancer SiHa cell line led to the induction of EMT and cell migration and invasion secondary to the activation of the Wnt/β-catenin pathway (132), indicating that the Tat/gp120-activated Wnt/β-catenin signaling pathway also contributes to EMT progression (132). Transcriptome analysis revealed that Tat/gp120-treated cells responded by upregulating keratin 17 and downregulating glycogen synthase kinase 3β, which promote the increase in vimentin and decrease in E-cadherin expression, respectively (i.e., EMT induction) (132).

Prolonged interactions (five days) of HIV-1 Tat and/or gp120 with HPV-16-immortalized oral (UM-SCC-47), cervical (CaSki), and anal (AKC-2) epithelial cells induce EMT and increased invasiveness of neoplastic cells (120). Furthermore, cells with Tat and/or gp120-induced EMT cells that co-expressed vimentin and E-cadherin and presented an intermediate/hybrid stage of EMT became poorly-adherent and easily detached (120). These cells also expressed CSC markers CD44 and CD133 and were more invasive, suggesting a specific role for hybrid EMT cells in cancer-associated metastasis.

HIV-1 Tat exposure also led to reductions in p53 expression, transcription of cell cycle inhibitors, and increased proliferation of HPV-18 infected cervical cancer HeLa cells (180). HIV-1 Tat also transactivated the HPV long control region and increased HPV18 E7 expression in HeLa cells (181).

Consistent with *in vitro* data, analysis of tissue samples from patients with cervical intraepithelial neoplasia (CIN) and cervical cancer who were infected with high-risk HPVs revealed significantly lower levels of E-cadherin and cytokeratin and higher levels of N-cadherin and vimentin expression in HIV-infected patients than those who were HIV-negative (132).

HIV-1 Tat inhibited epithelial differentiation and apoptosis of HPV-negative colorectal cancer cells (LIM1215 and LIM2537), increasing their invasiveness and tumorigenicity (182). HIV-1 Tat protein also induced EMT and increased invasiveness of HPV-negative lung epithelial (A549, H358) (183) and oral carcinoma (HSC-3) (120) cancer cell lines. These results suggest that HIV-1 Tat may promote the development of malignancy in both HPV-negative and HPV-infected neoplastic epithelial cells.

HIV-1 Tat-induced EMT may also promote epithelial neoplasia associated with other oncogenic viruses. For example, hepatitis C virus (HCV) coinfection with HIV-1 is detected quite frequently; approximately 30% of HIV-infected individuals may be coinfected with HCV (184–187). Primary liver cancer is more aggressive in patients with HIV-HCV coinfection than in those with HCV infection alone (188). HIV-1 Tat-induced EMT may develop in liver epithelial cells, thus accelerating HCV malignancy in coinfected individuals.

TGF-β expression remains persistently elevated in the blood of PLWH undergoing cART as well as those who are not (189–198). HIV-1-associated elevations of TGF-β contribute to the activation of EMT-associated profibrotic processes in various epithelial organs, including kidney, liver, and lung (199–208) (69, 209–214). It is possible that the elevated levels of TGF-β expression may induce EMT and ultimately increase invasiveness in precancer or cancer cells found in PLWH.

HIV-1 Tat induces TGF-β expression in epithelial cells, macrophages, and other cells (120, 121, 196, 215–218), which may contribute to its persistent elevation observed in PLWH. Persistent elevation of circulating TGF-β in PLWH undergoing cART may result from the continuous expression and secretion of HIV-1 Tat. Circulating TGF-β may reach the neoplastic tissues where it induces EMT and increased invasiveness. This may be one of the mechanisms underlying the persistence of HPV malignancy in PLWH treated with cART.

Taken together, the results of these studies suggest that direct interactions of extracellular HIV-1 Tat with neoplastic mucosal and other epithelial cells may lead to induction of EMT and increased invasiveness, leading to the acceleration of HPV-associated and HPV-negative malignancies (**Figure 2**). HIV-1 Tat-induced EMT may have a more rapid effect on the acceleration of HPV-infected neoplasia, given that oncogenic HPVs also induce EMT. For example, several groups reported that HPV-16 E6 and E7 oncoproteins induced the increased expression of MMP-2 (219–222) which is an enzyme that cleaves E-cadherin (223–225) and leads to the loss of adherens junctions and the induction of EMT (226). Both HIV- and HPV-induced EMT may have synergistic effects in the progression of HPV-associated neoplasia in PLWH.

## 6 HIV-1 Tat-mediated disruption of mucosal epithelium promotes herpes simplex virus and human cytomegalovirus spread

### 6.1 HIV-1-induced disruption of oral mucosal epithelium facilitates the spread of herpes simplex virus-1

HSV-1 and 2 may reactivate and replicate in the oral and genital epithelium of PLWH and can lead to ulcers and necrotic lesions (227–229). HSV-1 reactivation may occur despite ongoing cART (227, 230, 231). Although the increased risk of developing an HSV infection may be mediated in part by HIV-1-induced immune dysfunction, it may also be associated with direct and/or indirect molecular interactions between the two viruses.

As discussed in Section 4., interactions of HIV-1 Tat with oral epithelial cells can lead to the disruption of both adherens and tight junctions via the activation of the MAPK and NF-κB signaling pathways and the upregulation of MMP-9 (108, 109). HIV-1 Tat-mediated disruption of oral epithelial junctions facilitates the paracellular spread of HSV-1 (108).

The HSV-1 envelope gD binds to the cell adhesion protein nectin-1 (232), which is sequestered within intercellular junctions (**Figure 1B**); this localization plays a critical role in limiting HSV access to epithelial cells (233). HIV-1 Tat-mediated disruption of adherens junctions liberates nectin-1, thereby promoting HSV-1 gD binding to nectin-1. Thus, the extent of HSV-1 infection is substantially increased in disrupted compared to intact epithelial cells (108). Disrupted adherens junctions expose sequestered nectin-1 and thus accelerate the cell-to-cell spread of HSV-1. The introduction of anti-nectin-1 and anti-HSV-1 gD antibodies substantially blocked HSV-1 infection and cell-to-cell spread of the virus, indicating a critical role played by HIV-1 Tat-induced disruption of epithelial junctions and the liberation of nectin-1 in HSV infection and spread (108).

HSV-1 is latent in sensory neurons in healthy individuals with normal immune surveillance mechanisms (234). However, HIV-1 infection/acquired immunodeficiency syndrome (AIDS)-induced immune dysfunction leads to HSV-1 reactivation in neurons and the development of mucosal disorders (230). As noted above, HIV-1 Tat-mediated liberation of nectin-1 from cells with disrupted adherens junctions also promotes the spread of HSV-1 from neurons to epithelial cells. Thus, HIV-1 Tat-mediated disruption of intercellular junctions potentiates HSV-1 infection by facilitating paracellular and cell-to-cell spread of the virus within the mucosal epithelium and between epithelial cells and neurons. This mechanism may provide a mechanism to explain the rapid development of HSV-associated oral lesions in HIV-1-infected individuals.

### 6.2 HIV-1 Tat promotes human cytomegalovirus infection and spread in oral epithelium

HCMV can promote the development of oral mucosal lesions, retinitis, hepatitis, esophagitis, pneumonia, encephalopathy, and/or gastrointestinal inflammation (235–239). Nearly all HIV-1-infected individuals are co-infected with HCMV (10, 240). HCMV activation has also been reported in HIV-1-associated/AIDS disease (241).

HIV-1 Tat-induced disruption of tonsil epithelial junctions impaired their barrier function and promoted the paracellular spread of HCMV (242). Moreover, HIV-1 Tat-induced activation of NF-κB and MAPK signaling in these cells increases the extent of HCMV infection. NF-κB activation is critical for transactivation of the major immediate early HCMV promoter and the initiation of viral replication (243–245). MAPK activation also plays an important role in HCMV replication and the production of viral progeny (246).

Thus, HIV-1 Tat-induced disruption of the integrity of the oral mucosal epithelia may promote HCMV paracellular spread, which is critical for the initial entry of the virus in HCMV-negative individuals and the development of a systemic viral infection. Tat-induced HCMV paracellular spread is also important for transmitting the virus within the mucosal epithelium and the infection of intraepithelial and submucosal monocytes and macrophages, which may play an important role in promoting HCMV persistence (247–251). HIV-1 Tat-mediated induction of HCMV replication in epithelial cells through activation of NF-κB and MAPK signaling may contribute to viral amplification and dissemination, which lead to the development of mucosal lesions and other diseases.

HIV-1 Tat-induced disruption of epithelial junctions may promote infection and spread of other viruses that use tight junction proteins as receptors. For example, Hepatitis C Virus (HCV) uses occludin and claudins 1, 6, and 9 for entry (252–255). Reovirus entry requires JAM-1, occludin, and ZO-1 (256, 257); occludin is critical for coxsackievirus infection (258). HIV-1 Tat-induced disruption of epithelial tight junctions may expose these sequestered tight junction proteins to their cognate viruses, leading to their more rapid entry and spread.

## 7 HIV extracellular Tat plays a critical role in KS pathogenesis by promoting the proliferation, angiogenesis, and migration of endothelial cells

Kaposi’s sarcoma-associated herpesvirus (KSHV), known as human herpesvirus 8 (HHV8), is an etiological agent of Kaposi sarcoma (KS) (259), which is an AIDS-defining tumor with uncontrolled growth of endothelial cells with abnormal angiogenesis (260). KS may develop on the skin and/or oral/intestinal mucosa and can spread to lymph nodes and lungs (260).

HIV extracellular Tat plays a critical role in KS pathogenesis by synergizing with KSHV lytic proteins and thus promoting the proliferation of endothelial cells, their angiogenesis, and migration (30, 72, 261–271). HIV Tat interaction with endothelial cells leads to increased expression of the KSHV G protein-coupled receptor (vGPCR) and its signaling, which promotes viral infection and tumorigenicity (272–275). HIV Tat also promotes KSHV interleukin -6 (vIL-6)- and K1-induced angiogenesis (271, 276). HIV Tat-promoted increase of the oncogenic activity of KSHV proteins mostly occurs by Tat-associated regulation/modulation of the phosphatidylinositol 3-kinase/protein kinase B (PI3K/Akt) signaling pathway (271, 274, 277). Tat may also be involved in the expression of miRNAs, which increase angiogenesis, and thus accelerate KSHV-associated tumorigenesis (276).

In the pre-cART era, KS was approximately 20 – 50 fold more common in persons with HIV/AIDS than in the general population (278, 279). In PLWH, cART treatment markedly reduced the incidence of KS (280, 281). However, recent findings suggest that in PLWH the risk of KS is increasing despite the normalization of CD4 count and lack of HIV viremia (282–285). Increasing KS in PLWH with cART has been suggested as a wake-up call for research on HHV-8 (286).

The secretion of Tat in PLWH undergoing cART may contribute to the development of KS via Tat-induced proliferation, angiogenesis, and/or migration of HHV-8-infected endothelial cells. Moreover, results presented in other studies revealed that extracellular Tat promotes decreased expression of the tight junction proteins, claudin-1, claudin-5, and ZO-1 and/or ZO-2 in endothelial cells, leading to disruption of their intercellular junctions (189, 287–298). These changes may damage endothelial cells and increase the permeability of blood vessels, leading to endothelial barrier dysfunction, inflammation, and related disorders in the surrounding tissues (299, 300). This has been well characterized in studies of HIV-1 Tat-induced disruption of brain microvascular endothelial cells, which leads to impairment of the blood-brain barrier (BBB) and ultimately HIV-associated neurocognitive disorders (300–303). Tat-induced disruption of BBB may also result from Tat-mediated apoptosis of brain endothelial cells (304), as well as Tat-facilitated elevations in MMPs (297), endoplasmic reticulum (ER) stress, and mitochondrial dysfunction (304, 305).

KSHV-infected microvascular endothelial cells lose the capacity to express vascular endothelial cell markers (e.g., VE-cadherin, CD31, CD34, CD36, vascular endothelial growth factor receptor-3, and vascular endothelial growth factor C) and acquire expression of mesenchymal markers (fibroblast-specific protein-1, alpha 2 smooth muscle actin, type I/III collagen, vimentin, and N-cadherin), displaying endothelial-mesenchymal transition (EndMT) and invasive and migratory properties along with increased survival (306–313). KSHV-activated Notch-induced transcription factors Slug and ZEB1 were identified as critical for KSHV-induced EndMT (306, 307). TGFβ signaling pathway is also required for EndMT induction (314). However, KSHV did not activate the TGFβ signaling pathway in endothelial cells (306, 307). In persons diagnosed with HIV-KSHV coinfection, HIV-1 Tat-induced activation of the TGF-β signaling pathway might synergize with KHSV-induced Notch signaling, thereby accelerating migration and invasion of neoplastic endothelial cells.

## 8 Conclusions

HIV-1 Tat is expressed and secreted from HIV-infected cells of both ART-treated and untreated individuals. HIV-infected CD4^+^ lymphocytes, macrophages, and DC/LCs can migrate into the oral and cervical epithelium and secrete HIV-1 Tat protein in both neoplastic and non-neoplastic epithelial environments. HIV-1 Tat – epithelial cell interactions lead to activation of MAPK/NF-kB signaling followed by the disruption of tight and adherens junctions of epithelial cells and impaired barrier and immune/innate immune cell functions within the mucosal epithelium. HIV-1 Tat-activated MAPK and TGFβ-signaling in neoplastic epithelial cells induce EMT accompanied by the expression of the CSC markers CD133 and CD44, which amplifies the invasiveness of both HPV-infected and HPV-negative premalignant and malignant cells. HIV-1 Tat-induced disruption of epithelial junctions facilitates infection and spread of HSV-1 by liberating its otherwise sequestered gD receptor, nectin-1. HIV-1 Tat-activated NF-kB and MAPK signaling in epithelial cells promote the replication of HCMV and increase the production of viral progeny, respectively, leading to the spread of the virus within the oral mucosal epithelium. HIV Tat also plays a critical role in HHV-8-caused KS malignancy by promoting the proliferation, angiogenesis, and migration of neoplastic endothelial cells. Thus, HIV-1 Tat clearly contributes to the pathogenesis of HIV/AIDS despite the availability of cART. These findings emphasize the need for further investigation focused on the molecular mechanisms of Tat action in non-HIV-infected cells, including normal and neoplastic epithelial and endothelial cells. The development of a therapeutic vaccine targeting HIV-1 Tat may address some of these critical complications, including Tat-mediated disruption of epithelial junctions and induction of EMT.
